# The H3K27 demethylase, Utx, regulates adipogenesis in a differentiation stage-dependent manner

**DOI:** 10.1371/journal.pone.0173713

**Published:** 2017-03-20

**Authors:** Kazushige Ota, Kit I. Tong, Kouichiro Goto, Shuta Tomida, Akiyoshi Komuro, Zhong Wang, Kazuto Nishio, Hitoshi Okada

**Affiliations:** 1 Department of Biochemistry, Kindai University Faculty of Medicine, Osaka-sayama, Osaka, Japan; 2 Princess Margaret Cancer Centre, Campbell Family Institute for Breast Cancer Research, University Health Network, Toronto, Canada; 3 Department of Genome Biology, Kindai University Faculty of Medicine, Osaka-sayama, Osaka, Japan; 4 Department of Cardiac Surgery, Cardiovascular Research Center, University of Michigan, Ann Arbor, Michigan, United States of America; 5 Anti-Aging Center, Kindai University, Higashi Osaka, Osaka, Japan; University of Texas at Austin Dell Medical School, UNITED STATES

## Abstract

Understanding the molecular mechanisms that drive adipogenesis is important in developing new treatments for obesity and diabetes. Epigenetic regulations determine the capacity of adipogenesis. In this study, we examined the role of a histone H3 lysine 27 demethylase, the ubiquitously transcribed tetratricopeptide repeat protein on the X chromosome (Utx), in the differentiation of mouse embryonic stem cells (mESCs) to adipocytes. Using gene trapping, we examined *Utx*-deficient male mESCs to determine whether loss of Utx would enhance or inhibit the differentiation of mESCs to adipocytes. *Utx*-deficient mESCs showed diminished potential to differentiate to adipocytes compared to that of controls. In contrast, *Utx*-deficient preadipocytes showed enhanced differentiation to adipocytes. Microarray analyses indicated that the β-catenin/c-Myc signaling pathway was differentially regulated in *Utx*-deficient cells during adipocyte differentiation. Therefore, our data suggest that Utx governs adipogenesis by regulating c-Myc in a differentiation stage-specific manner and that targeting the Utx signaling pathway could be beneficial for the treatment of obesity, diabetes, and congenital *utx*-deficiency disorders.

## Introduction

Obesity is a major health concern worldwide [1–3]. Overconsumption of energy-rich foods facilitates fat deposition in adipose tissue [4]. Adipose tissue has a significant buffering capacity that permits it to adapt to excessive energy intake by changing the number and size of adipocytes [5,6]. The pathological accumulation of fat in the body leads to the risk of developing obesity-associated metabolic disorders [4,7]. Multiple processes of adipocyte differentiation including commitment to mesoderm, preadipocytes, and terminal differentiation into adipocytes are involved during adipocyte differentiation [8]. However, the mechanism of stage-specific regulation of adipogenesis is not still fully understood.

Histone methylation plays a pivotal role in diverse biological processes including adipogenesis [9,10]. The ubiquitously transcribed tetratricopeptide repeat protein on the X chromosome (Utx or Kdm6a) functions as a demethylase for a marker of gene repression, the histone H3 lysine 27 trimethylation (H3K27me3) [11–14]. Utx is ubiquitously expressed and indispensable for normal female development [15–17]. Utx is required for the maintenance of pluripotency in iPS cells [18], as well as for proper differentiation of mouse embryonic stem cells (mESCs) to mesenchymal and cardiac lineages [19]. Interestingly, *Utx* deficiency results in enhanced adipogenesis and decreased osteogenesis of mesenchymal stem cells (MSCs) [20]. These reports suggest that Utx has distinct roles during the process of adipocyte differentiation.

In this study, we examined the role of Utx in adipogenesis. In the differentiation of mESCs to adipocytes, *Utx*-deficient cells showed the lower number of terminally differentiated adipocytes than the control cells did. In contrast, the number of adipocytes was increased when *Utx*-deficient preadipocytes were differentiated. Microarray analysis showed that c-Myc is responsible for the distinct function of Utx in adipogenesis.

## Materials and methods

### Targeting Utx in mESCs

**Fig 1:**
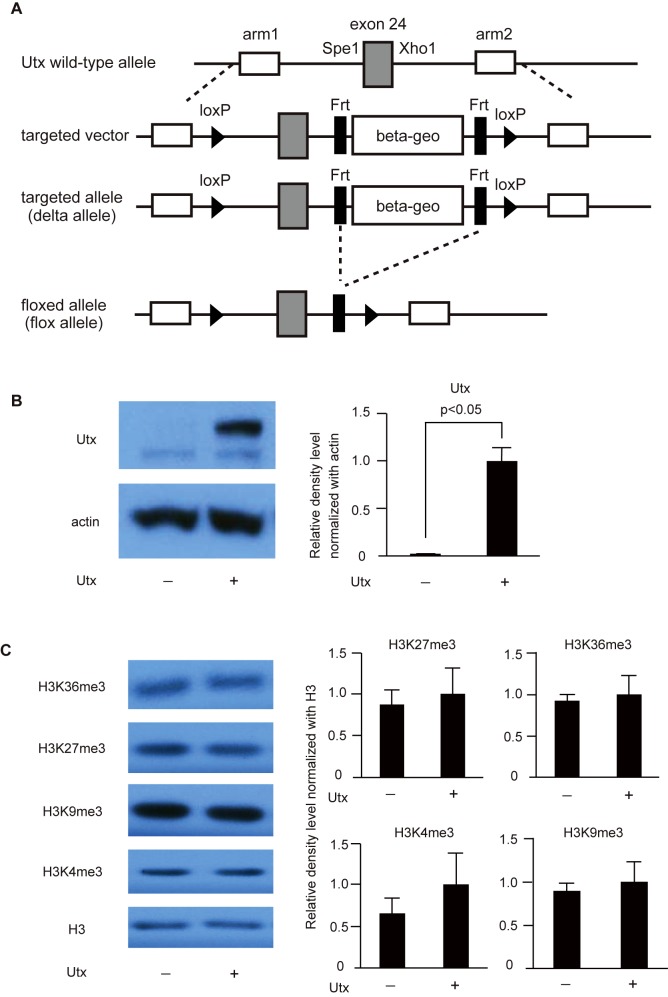
Construction and characterization of *Utx*-deficient mESCs. (A) Conditional gene targeting of the murine *Utx* locus. (B) Immunoblotting for Utx and β-actin in *Utx*-deficient and control mESCs. A representative result from three independent mESC clones is shown. Band densities of Utx and β-actin were quantified by ImageQuant TL ver. 8.1 and normalized to β-actin. The data are presented relative to the signal of control mESCs. (C) Immunoblotting for H3, H3K4me3, H3K9me3, H3K27me3, and H3K36me3 in mESCs. A representative result from three independent mESC clones is shown. Band density of histones was quantified by ImageQuant TL ver. 8.1 (GE healthcare life sciences, Pittsburgh, PA, USA), and data are shown by the relative value of histone methylations normalized to H3.

The *Utx*-targeting vector was constructed such that the β-geo cassette was flanked by FRT (flippase recognition target) sequences (black rectangles), and exon 24 of *Utx* was flanked by loxP sequences (black triangles); a portion of the wild-type murine *Utx* locus including exon 24 (grey rectangle) and a 0.5 kb SpeI-XhoI fragment are depicted alongside the targeting vector in Fig 1A. The conditional targeting vector was used to generate three independent ES cell lines 129/Ola (a kind gift from Dr. Tak W. Mak, Princess Margaret Cancer Centre, Campbell Family Institute for Breast Cancer Research, University Health Network). The β-geo cassette was removed *in vitro* by expressing a flippase recombinase. Homologous recombination in mESCs was confirmed by Southern blot analysis.

The mESCs were cultured in Dulbecco’s modified Eagle medium (DMEM; Invitrogen, Burlington, ON, Canada) containing 20% fetal bovine serum (Hyclone, GE Healthcare, Amersham, Buckinghamshire, U.K.), leukemia inhibitory factor (LIF; Merck Millipore, Darmstadt, Germany), L-glutamate (Sigma-Aldrich, St Louis, MO, USA), sodium pyruvate (Sigma-Aldrich, St Louis, MO, USA), penicillin/streptomycin (Sigma-Aldrich, St Louis, MO, USA), and 2-β-mercaptoethanol (Sigma-Aldrich, St Louis, MO, USA) on 0.5% gelatin-coated dishes at 37°C with 5% CO_2_.

### Adipogenic differentiation of mESCs

Differentiation of mESCs was induced using the Mouse Embryonic Stem Cell Adipogenesis Kit (SCR100; Merck Millipore, Darmstadt, Germany). In this method, 0.1 μM retinoic acid (RA), 20 nM Triiodothyronine (T3), and 850 nM insulin were used to induce the differentiation to adipocytes [21,22]. After removing feeder cells, 3 × 10^6^ mESCs were cultured in 10 mL embryonic body Formation Medium on a non-adhesive 10 cm Petri dish at 37°C with 10% CO_2_ for 2 days without LIF. RA (Inducer A Solution) was added to the culture for an additional 3 days to induce efficient differentiation into adipocytes (37°C with 10% CO_2_). Subsequently, for an additional 21 days, 10–20 EBs were cultured in 1 mL of Adipocyte Differentiation Medium supplemented with T3 and insulin on 0.1% gelatin-coated 24-well plates at 37°C with 10% CO_2_. For the inhibition of c-Myc, 25 μM 10058-4F (Sigma-Aldrich, St Louis, MO, USA) was added during differentiation from mESCs to adipocytes.

### Culture and differentiation of 3T3-L1 cells to adipocytes

3T3-L1 cells were purchased from ATCC and cultured in DMEM supplemented with 10% calf serum (Invitrogen, Burlington, ON, Canada) and penicillin/streptomycin at 37°C with 5% CO_2_. For adipocyte differentiation, two days after cells grew to confluency in 10% calf serum/DMEM, they were cultured in DMEM containing 10% fetal bovine serum (Invitrogen, Burlington, ON, Canada), 1 μg/mL insulin (Sigma-Aldrich, St Louis, MO, USA), 0.5 mM isobutylmethylxanthine (Sigma-Aldrich, St Louis, MO, USA), and 10 μM dexamethasone (Sigma-Aldrich, St Louis, MO, USA). Two days after the culture, cells were cultured with 10% fetal bovine serum/DMEM supplemented with 1 μg/mL insulin. Two days after culturing cells in insulin, to differentiate them to adipocytes, the cells were cultured with 10% fetal bovine serum/DMEM by changing the media every alternate day. For the inhibition of c-Myc, 50 μM 10058-4F was added during adipocyte differentiation.

### Lentiviral vector-mediated stable knockdown

The pLKO.1 puro lentiviral vector and control scrambled shRNA were purchased from Addgene (Addgene # 8453 and #1864) (Addgene, Cambridge, MA, USA). The mouse Utx target sequence was cloned into pLKO.1 vector. To generate lentivirus, pLKO.1-Utx with pMD2.G and psPAX2 (Addgene #12259 and #12260) were transfected into HEK293 cells. The target sequence was shown in S1 Table.

### Total RNA isolation and qRT-PCR analysis

Total RNA was extracted using the Trizol reagent (Thermo Fisher Scientific, MA, USA). First-strand cDNA was synthesized using the iScriptTM cDNA Synthesis Kit (Bio-Rad, Hercules, CA, USA), followed by PCR with primers specific for target genes. Quantitative real time-PCR (qRT-PCR) was performed using the Power SYBR Green PCR Master Mix (Thermo Fisher Scientific, MA, USA), and the data were collected using the ABI Prism 7900HT Fast Real-time PCR system (Thermo Fisher Scientific, MA, USA). Specificity of the signals was confirmed by a dissociation curve analysis (containing a single peak), followed by electrophoresis of the PCR product. The results are shown as mean mRNA levels normalized to the amount of Actb or Gapdh mRNA ± SE from three independent experiments with three different clones or triplicate of qPCR with one clone as indicated. The primers used in the study were shown in S1 Table.

### Immunoblotting assay

Whole cell lysates were prepared with CHAPS lysis buffer supplemented with 40 mM HEPES (pH 7.5), 120 mM NaCl, 1 mM EDTA, 0.3% CHAPS, 50 mM NaF, 1.5 mM Na_3_VO_4_, 10 mM glycerophosphate, 10 mM pyrophosphate, 1 mM PMSF, protease inhibitor cocktail (Roche Diagnostics, Basel, Switzerland), and phosphatase inhibitor cocktail (Roche Diagnostics, Basel, Switzerland). Triton extraction buffer was used to harvest the histones. Triton extraction buffer is composed of phosphate-buffered saline (PBS; pH 7.4) containing 0.5% Triton X-100, 5 mM sodium butyrate, and protease inhibitor cocktail (Roche Diagnostics, Basel, Switzerland). Lysates were separated by SDS-PAGE and transferred to a PVDF membrane (Merck Millipore, Darmstadt, Germany). The membranes were blocked in blocking buffer (5% w/v BSA or 5% w/v skim milk, 0.05% v/v Tween-20 in TBS), and subsequently incubated with primary and secondary antibodies in the blocking buffer. ECL blotting reagents (GE Healthcare, Buckinghamshire, UK) were used to detect the immunoreactive proteins. Antibodies recognizing the following proteins were used: Utx (Sigma-Aldrich, St Louis, MO, USA), β-actin (Santa Cruz Biotechnology, Texas, USA), H3 (Abcam, Cambridge, UK), H3K4me3 (Abcam, Cambridge, UK), H3K9me3 (Abcam, Cambridge, UK), H3K27me3 (Abcam, Cambridge, UK), and H3K36me3 (Abcam, Cambridge, UK).

### Oil Red O staining

Cells were fixed on the tissue culture plate for 10 min with 10% formalin. After fixation, cells were washed twice with PBS and once with 60% isopropanol. Cells were stained with Oil Red O (Sigma-Aldrich, St Louis, MO, USA) for 15 min and then washed twice with PBS.

### Microarray assay

The preparation of total RNA from mESCs and differentiated adipocytes was carried out using the Trizol reagent (Thermo Fisher Scientific, MA, USA). Illumina whole mouse genome arrays (Mouse Ref-8 v2 Beadchip) were used for the microarray chip. The microarray analysis, including hybridization and data collection, was performed by Illumina. The transcript assignments were based on the National Center for Biotechnology Information Reference Sequence (NCBI RefSeq) database (Build 36, Release 22). After importing compressed library files, we used the lumi package with default settings from R-Bioconductor for subsequent analyses, including normalization. After calculating the expression ratios before and after adipocyte differentiation, candidate genes with a ratio exceeding four standard deviations (SDs) were selected simultaneously in two different clones. The upregulated or downregulated genes were then analyzed by Ingenuity pathway analysis software (IPA; Qiagen, Hilden, Germany) to predict upstream regulators. Gene set enrichment analyses (GSEA) were performed with a GSEAv2.0 package (http://www.broad.mit.edu/gsea) with the following settings: a signal-to-noise metric for gene ranking, and 1000 permutations of gene sets. We used the established collections of gene sets (c2, c4, c5, and c6) provided by Molecular Signatures Database (MSigDB, http://www.broad.mit.edu/gsea/msigdb). The nominal p value for ANOVA analysis was set as p<0.05 and further adjusted by FDR (Step Up, FDR <0.25).

### Statistical analysis

All data are presented as mean ± SE. All statistical analyses except the microarray analyses were done using the Student’s *t*-test or Welch’s correction. A p-value of < 0.05 was considered significant in all tests.

## Results

### Generation of *Utx*-deficient mESCs

To examine whether Utx is involved in adipogenesis, we generated *Utx*-deficient mESCs (Fig 1A). *Utx*-null female mESCs are unable to differentiate into mesoderm and *Utx*-null female embryos arrest at E10.5 [16,19]. Male mESCs have one copy of Utx and one copy of a related demethylase, Uty, on the Y-chromosome. *Utx*-deficient male zygotes survive until birth, demonstrating that Uty can compensate for Utx [15–17]. Therefore, the roles of Utx during adipocyte differentiation can be investigated using *Utx*-deficient male mESCs. Through expression of the flippase recombinase, we removed the gene-trapping element of the construct, restoring normal expression of the target gene. We generated three independent clones of *Utx*-deficient mESCs. Endogenous Utx protein expression was mostly undetected in *Utx*-deficient mESCs, whereas in control mESCs, a band of Utx is detected after expression of flippase (Fig 1B). Consistent with previous reports [16], *Utx* deficiency did not affect the global levels of H3K4me3, H3K9me3, H3K27me3, or H3K36me3 (Fig 1C, left). To further confirm this, we measured the band density of the histone modifications in three independent clones. The intensity of the bands showed no differences between control and *Utx*-deficient mESCs (Fig 1C, right). These data confirm that our targeted mESCs showed diminished *Utx* expression and retained normal levels of lysine modifications in histone H3.

### *U*tx deficiency in mESCs impairs adipocyte differentiation

**Fig 2:**
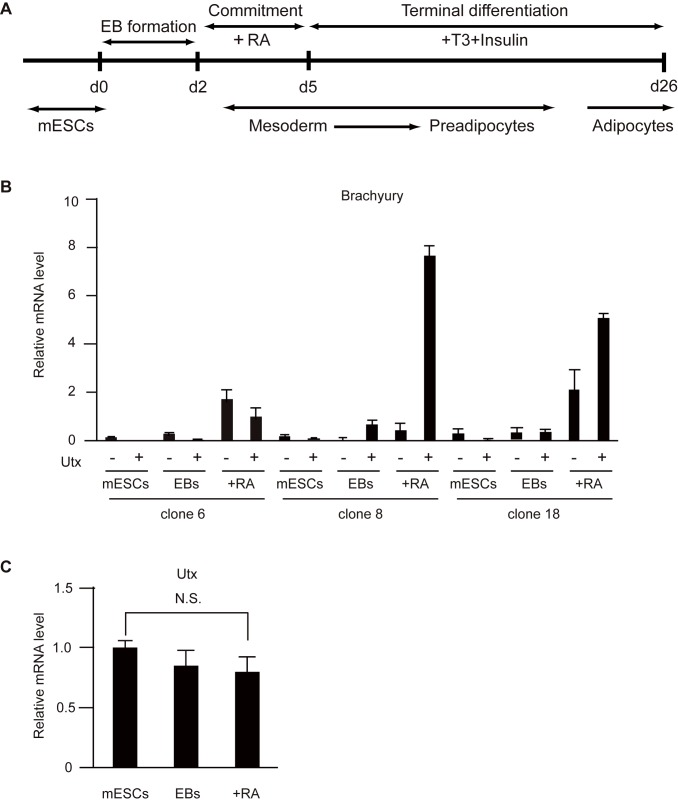
*Utx*-deficient mESCs showed some potential to differentiate into mesoderm. (A) Scheme of the differentiation assay of mESCs to adipocytes. (B, C) mRNA levels of *Brachyury* (B), *Utx* (C) normalized to *β-actin* in mESCs and adipocytes. The experiments were performed independently with three clones, and the results are expressed as mean ± SE (n = 3); *p<0.05.

To examine roles of Utx in adipogenesis, we performed adipocyte differentiation assays using control and *Utx*-deficient mESCs (Fig 2A). As the protocol, we obtained mesoderm with the potential to differentiate into adipocytes, after treatment with RA from day 2 to day 5, to form EBs [21,22]. The levels of *Brachyury* were evaluated to determine whether our *Utx*-deficient mESCs could differentiate into mesoderm in EBs (Fig 2B). Depending on the clone, *Brachyury* was differentially expressed in *Utx*-deficient EBs compared to that in controls. Our *Utx*-deficient male mESCs established by gene-trapping show some potential to differentiate into mesoderm because of residual Utx expression in contrast to the previous papers [16,17,23].

**Fig 3:**
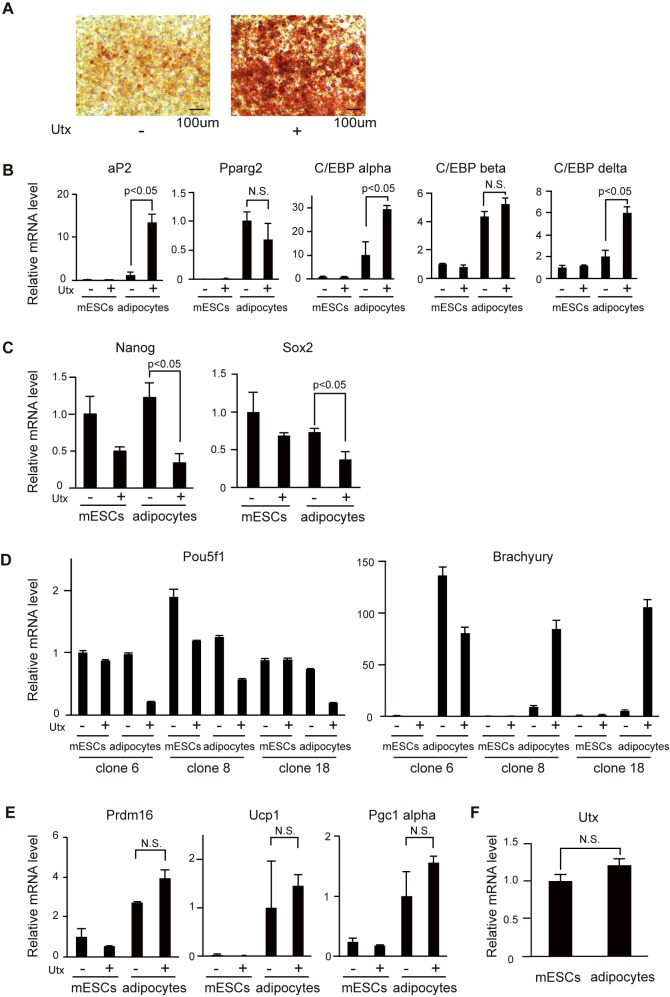
*Utx*-deficient mESCs showed diminished differentiation to adipocytes. (A) Oil-red O staining of adipocytes. Representative results from three independent mESC clones are shown. (B-F), mRNA levels of *aP2*, *Pparg2*, *C/EBP alpha*, *C/EBP beta*, *C/EBP delta* (B)*; Nanog*, *Sox2* (C); *Pou5f1*, *Brachyury* (D); *Prdm16*, *Ucp1*, *Pgc1 alpha* (E); *Utx* (F) normalized to *β-actin* in mESCs and adipocytes. The experiments were performed with three independent clones, and the results are expressed as mean ± SE (n = 3); *p<0.05.

It has not been investigated whether *Utx* deficiency affects terminal adipocyte differentiation. Because our *Utx*-deficient mESCs with gene trapping have some potential to induce the genes responsible for mesoderm, induction of adipocytes with insulin and T3 after the RA-induced differentiation was performed. All of the clones of *Utx*-deficient cells after induction (described as adipocytes) showed a lower number of Oil-red O positive cells (Fig 3A) and decreased expression of the adipocyte marker, *aP2* (Fig 3B). The expression levels of *C/EBP alpha* and *C/EBP delta* were also significantly lower in *Utx*-deficient adipocytes (Fig 3B). *Pparg2* and *C/EBP beta* were induced at similar levels in control and *Utx*-deficient adipocytes (Fig 3B). The expression of *Nanog*, *Sox2*, and *Pou5f1* was consistently higher in *Utx*-deficient adipocytes, suggesting that some cells remained undifferentiated in the *Utx*-deficient cells (Fig 3C and 3D). The expression of *Brachyury* was differentially induced in *Utx*-deficient adipocytes depending on the clones (Fig 3D). Recently, Utx has been reported to promote the expressions of genes responsible for thermogenesis in brown adipose tissue [24,25]. In our experiment, *Utx* deficiency did not affect the expression of *Prdm16*, *Pgc1 alpha*, or *Ucp1*, which are critical for thermogenesis in brown adipose tissue (Fig 3E). During differentiation, *Utx* expression in adipocytes was comparable to that in mESCs (Fig 3F). Our results indicate that RA-induced differentiation in *Utx*-deficient cells has lower potential to differentiate to adipocytes, and that Utx is required for adipogenesis during differentiation of mESCs to adipocytes.

### Gene expression profile of control and *Utx*-deficient cells

**Fig 4:**
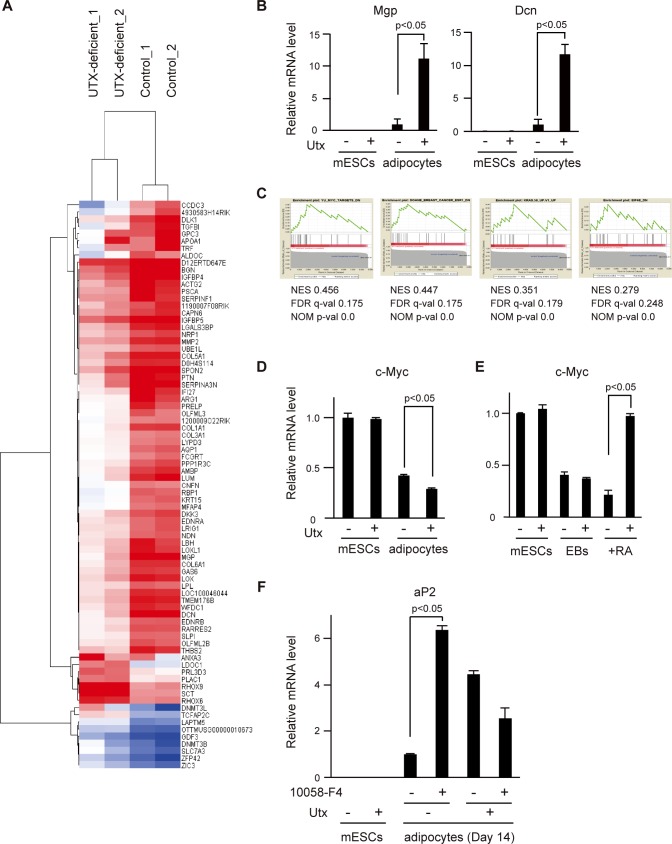
Effects of Utx deficiency on gene expression in mESCs and adipocytes. (A) Heat map visualizations of the 79 genes that are differentially expressed in *Utx*-deficient and control mESCs and adipocytes. Two clones were used for each group. Data for the heat maps are normalized using average linkage clustering on entities and represent median-centered log-transformed values. Red and blue correspond to high and low expression, respectively, compared with the experiment-wide median. (B) mRNA levels of *Mgp* and *Dcn* normalized to *β-actin*. The experiments were performed independently with three clones, and the results are expressed as mean ± SE (n = 3); *p<0.05. (C) GSEA enrichment score curves described with two sets of adipocytes from the Molecular Signatures Database (MSigDB). The graph at the bottom of each panel represents the ranked, ordered, non-redundant list of genes. Vertical black lines indicate the position of genes from the studied gene set in the ordered, non-redundant data set. The green curve corresponds to the enrichment score (ES) curve, which is the running sum of the weighted enrichment score obtained with the GSEA software. NES, normalized enrichment score: FDR, false discovery rate; NOM, nominal. (D, E) mRNA levels of *c-Myc* normalized to *β-actin* in (D) mESC and adipocytes, (E) mESCs, EBs, and RA-induced differentiation. The experiments were performed independently with three clones, and the results are expressed as mean ± SE. (n = 3) *p<0.05. (F) mRNA levels of *aP2* normalized to *Gapdh* in *Utx*-deficient or control mESCs and adipocytes in the presence of 10058-F4. The representative result is shown as mean ± SE. (n = 3) *p<0.05.

To investigate the genes affected by the loss of *Utx*, we analyzed gene expression profiles of control and *Utx*-deficient mESCs and adipocytes. We performed microarray analyses of two different sets of the mESC clones, clone 8 and clone 18. After calculating the expression ratio before and after adipocyte differentiation, candidate genes with a ratio exceeding 4 SD were selected. Both data sets of mESCs and adipocytes were clustered in a heat map and a tree diagram (Fig 4A). The major clusters, including genes upregulated in control cells (63 genes) and those upregulated in *Utx*-deficient cells (16 genes), are shown in Fig 4A. Validation of the microarray data was performed with qRT-PCR for *Mgp* and *Dcn*, the genes with the largest changes in expression between the sets (Fig 4B).

To study the genetic networks regulated by Utx, we performed a pathway analysis on the gene sets exceeding 4 SD with IPA. The gene sets upregulated in *Utx*-deficient cells suggest that critical genes for pluripotency, such as *Nanog*, *Pou5f1*, and *Lamtor3*, are involved in Utx signaling (S2 Table). The gene sets upregulated in control cells suggest that *Htt*, *α-catenin*, and *Fas* are the most significant upstream regulators of Utx (S3 Table).

We also performed GSEA with the gene sets of adipocytes to find the sets regulated by Utx. This revealed similar gene expression patterns involving c-Myc, ER1, k-Ras, and E1F4E signaling pathways in the data set from *Utx*-deficient adipocytes (Fig 4C). Both GSEA and IPA analyses suggested increased expression of genes in the Wnt/β-catenin/c-Myc axis, which includes α-catenin and c-Myc [26–29]. First, we examined the expression of *c-Myc* during the differentiation from mESCs to adipocytes in *Utx*-deficient and control cells. The expression of *c-Myc* was significantly upregulated in *Utx*-deficient adipocytes while the expression of *c-Myc* was downregulated in *Utx*-deficient cells after RA-induced differentiation (Fig 4D and 4E). Secondly, to determine whether Utx affects adipogenesis by regulating c-Myc function, we examined adipocyte differentiation of *Utx*-deficient mESCs with a Myc inhibitor, 10058-F4. The expression level of *aP2* was significantly higher in the adipocytes differentiated from *Utx*-deficient mESCs in the presence of 10058-F4 compared to those in the untreated *Utx*-deficient adipocytes (Fig 4F). Taken together, our findings indicate that c-Myc plays a significant role in Utx-mediated adipogenesis.

### *Utx* deficiency enhances differentiation of preadipocytes to adipocytes

**Fig 5:**
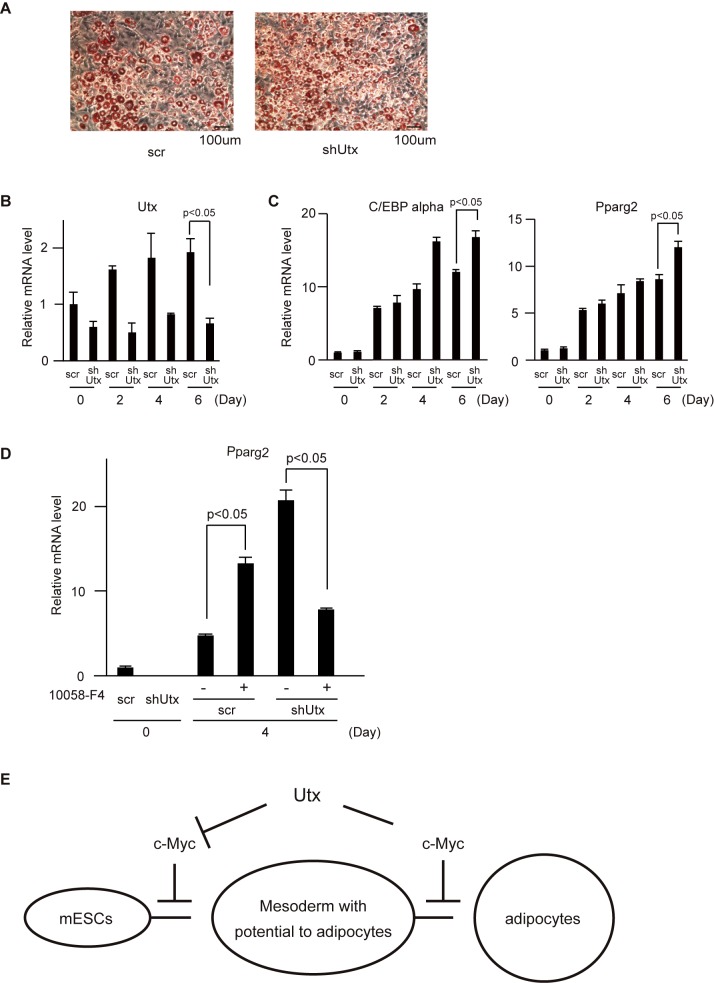
Knockdown of *Utx* in 3T3-L1 cells resulted in an enhancement of adipocyte differentiation. (A) Oil-red O staining of adipocytes. Representative results from three independent experiments are shown. mRNA levels of (B) *Utx*, (C) *C/EBP alpha*, *Pparg2*, (D) *Pparg2* normalized to *β-actin*. The experiments were performed independently three times in triplicates and the representative results are shown and expressed as mean ± SE. (n = 3) *p<0.05. (E) Schematic model of Utx during differentiation of mESCs to adipocytes.

Differentiation of mESCs to adipocytes involves multiple differentiation steps: formation of mesoderm, mesenchymal lineage, and preadipocytes. In our experiment, each differentiation stage was classified as shown in Fig 2A. Although *Utx* deficiency resulted in impairment to differentiate to adipocytes, several critical genes for the adipocyte lineage were induced in *Utx*-deficient adipocytes to levels comparable to those in control cells. In a recent study, *Utx* deficiency in MSCs resulted in enhanced adipocyte differentiation [20]. Therefore, we hypothesized that Utx functions as a positive or negative regulator, depending on a differentiation stage. To examine whether *Utx* deficiency enhances or inhibits adipocyte differentiation in preadipocytes, knockdown of *Utx* was performed in 3T3-L1 cells (preadipocytes) to differentiate them to the white adipocyte lineage. In contrast to mESCs differentiation, *Utx* depletion in 3T3-L1 cells enhanced adipocyte differentiation (Fig 5A). The efficiency of *Utx* knockdown was confirmed by qRT-PCR (Fig 5B). The expression of *C/EBP alpha* and *Pparg2* confirmed an enhancement of terminal adipocytes differentiation (Fig 5C). To examine the contribution of c-Myc during differentiation from preadipocytes to adipocytes, we differentiated 3T3-L1 depleted for *Utx* in the presence of 10058-F4 (Fig 5D). Inhibition of c-Myc in *Utx*-depleted 3T3-L1 cells resulted in the inhibition of adipocyte differentiation (Fig 5D). In contrast, control 3T3-L1 cells with the Myc inhibitor enhanced adipocyte differentiation (Fig 5D), which is consistent with the previous report [30]. These results indicate that Utx negatively regulates the differentiation of preadipocytes to adipocytes by enhancing activation of c-Myc.

## Discussion

In this study, we established *Utx*-deficient mESCs with gene trapping and evaluated their potential to differentiate to adipocytes. *Utx*-deficient mESCs showed some potential to differentiate to mesoderm. Moreover, *Utx*-deficient cells after RA-induced differentiation did not differentiate to adipocytes, showing that Utx is required for proper commitment to mesoderm with potential to differentiate to adipocytes. Conversely, *Utx* knockdown in preadipocytes showed enhanced differentiation to adipocytes. Therefore, Utx differentially regulates adipogenesis depending on the stages of differentiation. Gene expression analysis revealed that c-Myc may be regulated by Utx.

### Distinct roles of Utx during adipocyte differentiation

Our results are the first to demonstrate an impairment of adipocyte differentiation in *Utx*-deficient male mESCs. *Utx*-deficient male mice can survive over one year, indicating that Utx is dispensable for male mESC development *in vivo*, despite partial lethality [17]. Although a previous study showed that *Utx*-deficient male mESCs have mesoderm defects [16,17,23], our results suggest that *Utx*-deficient mESCs can differentiate to mesoderm (Fig 2B). Recently, *Utx* deficiency has been reported to induce a subset of critical genes associated with mesoderm differentiation including *Brachyury* [15,16]. In our *Utx*-deficient male mESCs, *Brachyury* was induced though its expression level was dependent on a clone. One possibility is that the time and concentration needed to react with RA to form EBs are critical conditions for mesoderm differentiation because RA inhibits mesoderm differentiation by repressing *Brachyury* expression [31]. Another possibility is that our *Utx*-deficient mESCs with gene trapping still express Utx, which could function to induce some of the genes needed for mesoderm differentiation. However, our *Utx*-deficient mESCs showed impairment to differentiate to adipocyte, consistent with recent studies that Utx is required for proper induction of mesoderm [16,17,23].

In preadipocytes, knockdown of *Utx* resulted in an enhancement of adipocyte differentiation. The same effect was observed after Utx knockdown in MSCs [20]. Our results uncovered that Utx inhibits adipocyte differentiation after commitment to preadipocytes. In our method, it may be difficult to obtain enough brown adipose cells, as shown previously [32]. We cannot discuss differentiation and function between white adipose cells and brown adipose cells with *Utx* deficiency, although the genes responsible for thermogenesis were equally induced in *Utx*-deficient cells as well as control cells (Fig 3D). Collectively, we propose distinct functions of Utx in a differentiation stage-dependent manner (Fig 5E).

### Networks of genes and signaling pathways dependent on Utx

Our microarray data analyses have identified potential target genes and signaling pathways regulated by Utx. We focused on the Wnt/β-catenin/c-Myc pathway which involves α-catenin and c-Myc because it is known to be one of the critical pathways of adipocyte differentiation [26–29]. α-catenin counteracts β-catenin, a key component of the canonical Wnt signaling pathway [26,28]. Recently, Utx has been reported to induce Wnt and DKK gene families by removing repressive H3K27me3 marks in endoderm differentiation [33]. The expression profile of c-Myc in differentiation stages of *Utx*-deficient cells implies that Utx regulates the Wnt signaling pathway during differentiation from mESCs to adipocytes. Furthermore, our experiments with the Myc inhibitor suggest that Utx enhances adipogenesis in mESCs by inhibiting c-Myc and represses adipogenesis in preadipocytes by activating c-Myc (Fig 5E). Since Utx is known to interact with c-Myc [34], we speculate that Utx may modulate transcriptional activation of c-Myc during adipocyte differentiation in a differentiation stage-dependent manner.

### Clinical significance

Commitment reflects hyperplasia (cell number) of adipose tissue and is mainly dependent on genetic background, whereas terminal differentiation to adipocytes affects hypertrophy (cell size) of adipose tissue and is mainly dependent on diet [6]. It is reported that adult-onset obesity is mainly caused by hypertrophy, whereas young-onset obesity is due to both hyperplasia and hypertrophy [5]. Because our data showed that Utx promotes commitment from mESCs to mesoderm with potential to adipocytes, Utx may have implications in glucose and fat metabolism in childhood. Additionally, because Utx inhibits the differentiation of preadipocytes to adipocytes, Utx in adipose tissues may reflect potential for adipose tissue development/expansion in adults on diets featuring excess energy intake. With the knowledge of handling and storing of nutrition, the tendency towards obesity and diabetes may be predicted and managed. Further studies using mouse and human samples will verify our hypothesis.

In conclusion, our data indicate that Utx is required for proper differentiation of mESCs to adipocytes. By identifying Utx target genes at different developmental stages during adipocyte differentiation, it may be possible to treat metabolic syndromes and improve the outcomes of their associated diseases.

## Supporting information

S1 Table Sequences of Utx knockdown and primers for RT-qPCR. (DOC)

S2 Table Upstream regulators in the gene set upregulated in *Utx*-deficient cells. (XLS)

S3 Table Upstream regulators in the gene sets upregulated in control cells. (XLS)
