# Correction: The H3K27 demethylase, Utx, regulates adipogenesis in a differentiation stage-dependent manner

**DOI:** 10.1371/journal.pone.0176424

**Published:** 2017-04-19

**Authors:** The PLOS ONE Staff

The arrows are missing from Fig 5E. The publisher apologizes for the error. Please see the corrected Fig 5 here.

**Fig 5:**
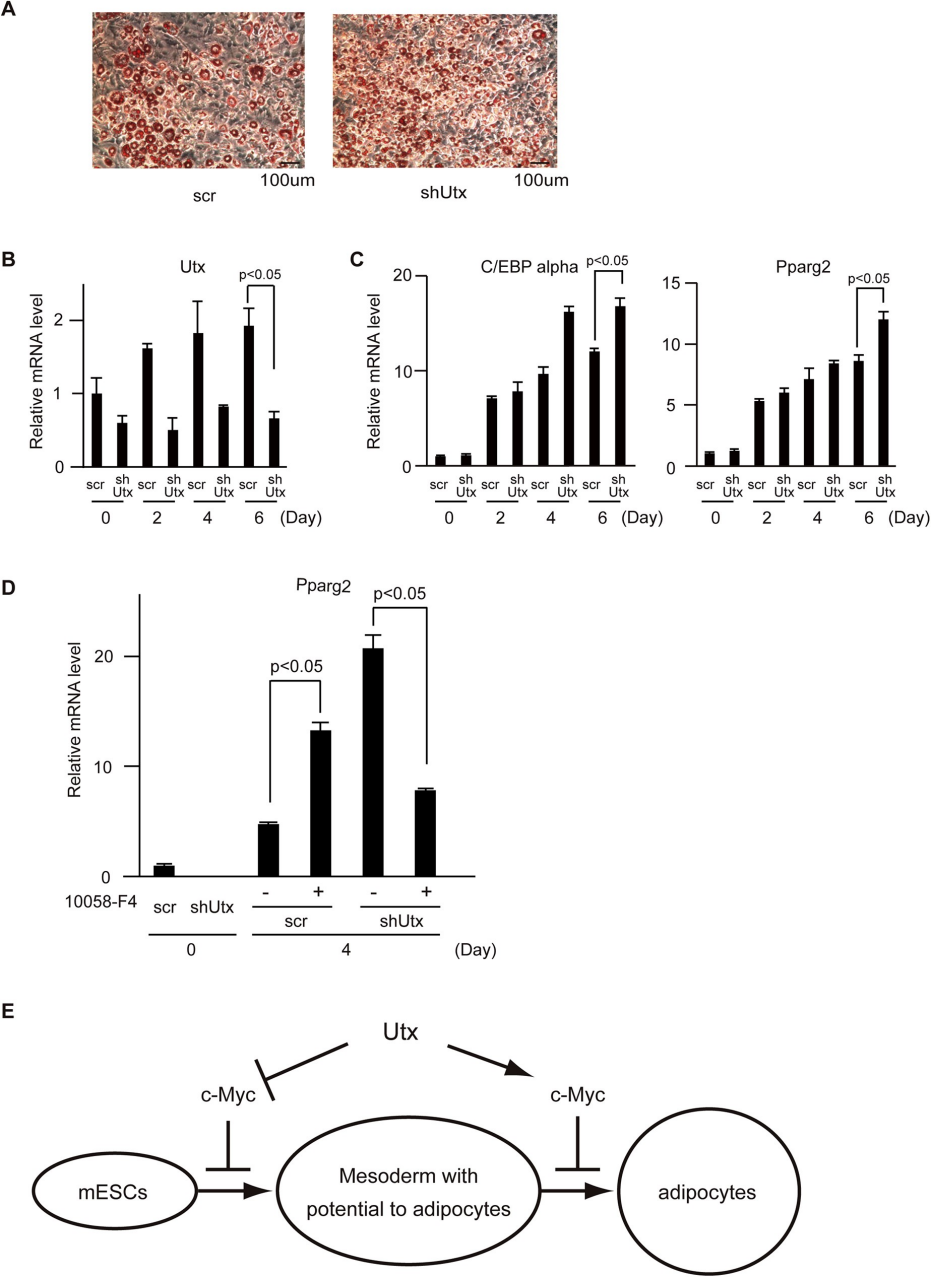
Knockdown of *Utx* in 3T3-L1 cells resulted in an enhancement of adipocyte differentiation. (A) Oil-red O staining of adipocytes. Representative results from three independent experiments are shown. mRNA levels of (B) *Utx*, (C) *C/EBP alpha*, *Pparg2*, (D) *Pparg2* normalized to *β-actin*. The experiments were performed independently three times in triplicates and the representative results are shown and expressed as mean ± SE. (n = 3) *p<0.05. (E) Schematic model of Utx during differentiation of mESCs to adipocytes.
