# Supplementary material for: The H3K27 demethylase, Utx, regulates adipogenesis in a differentiation stage-dependent manner
Source: PLoS One. 2017 Mar 20;12(3):e0173713. doi: 10.1371/journal.pone.0173713 (PMC5358847; doi:10.1371/journal.pone.0173713)
Supplement: S1 Table — (DOC) [file pone.0173713.s001.doc]

**Supporting Information table 1**

**Utx shRNA target sequence**: GAGCCAAGGAAATTCATTT

***Primers for real-time RT-PCR***

| **Name** | **Forward** | **Reverse** |
| --- | --- | --- |
| *Actb* | GGCTGTATTCCCCTCCATCG | CCAGTTGGTAACAATGCCATGT |
| *Brachyury* | GCTGGATTACATGGTCCCAAG | GGCACTTCAGAAATCGGAGGG |
| *Utx* | ATCCCAGCTCAGCAGAAGTT | GGAGGAAAGAAAGCATCACG |
| *pparg2* | TGCCTTGCTGTGGGGATGTCTC | CCTCGCCTTGGCTTTGGTCAG |
| *C/EBP alpha*, | GGTGCGGGCAAAGCCAAGAAGT | GCCCCGCAGCGTGTCCAGTT |
| *C/EBP beta* | GGCGCGAGCGCAACAACATC | GCTCGGGCAGCTGCTTGAACAA |
| *C/EBP delta* | CGACTTCAGCGCCTACATTGA | CTAGCGACAGACCCCACAC |
| *Nanog* | TCTTCCTGGTCCCCACAGTTT | GCAAGAATAGTTCTCGGGATGAA |
| *Pou5f1* | AGAGGATCACCTTGGGGTACA | CGAAGCGACAGATGGTGGTC |
| *Sox2* | GCGGAGTGGAAACTTTTGTCC | CGGGAAGCGTGTACTTATCCTT |
| *Ucp1* | AGGCTTCCAGTACCATTAGGT | CTGAGTGAGGCAAAGCTGATTT |
| *Pgc1 alpha* | TGCGGGATGATGGAGACA | GCGAAAGCGTCACAGGTGTA |
| *Prdm16* | CAGCACGGTGAAGCCATTC | GCGTGCATCCGCTTGTG |
| *Mgp* | GGCAACCCTGTGCTACGAAT | CCTGGACTCTCTTTTGGGCTTTA |
| *Dcn* | TCTTGGGCTGGACCATTTGAA | CATCGGTAGGGGCACATAGA |
| *c-Myc* | TCCTCCCCACGGGCCAGCC | GGCAGGGGTTTGCCTCTTCT |
| *Gapdh* | aacgaccccttcattgac | tccacgacatactcagcac |
|  | | |
